# *Staphylococcus epidermidis* and *Staphylococcus haemolyticus*: Molecular Detection of Cytotoxin and Enterotoxin Genes

**DOI:** 10.3390/toxins7093688

**Published:** 2015-09-14

**Authors:** Luiza Pinheiro, Carla Ivo Brito, Adilson de Oliveira, Patrícia Yoshida Faccioli Martins, Valéria Cataneli Pereira, Maria de Lourdes Ribeiro de Souza da Cunha

**Affiliations:** Departamento de Microbiologia e Imunologia, Instituto de Biociências, UNESP—Univ Estadual Paulista, Botucatu, SP 18618-970, Brasil; E-Mails: luizapinheiro@ibb.unesp.br (L.P.); adilsonoliveiralp@ig.com.br (A.O.); patríciayf@yahoo.com.br (P.Y.F.M.); valeriacataneli@gmail.com (V.C.P.)

**Keywords:** *Staphylococcus epidermidis*, *Staphylococcus haemolyticus*, enterotoxins, cytotoxins

## Abstract

Although opportunistic pathogens, coagulase-negative staphylococci (CoNS), including *Staphylococcus epidermidis* and *Staphylococcus haemolyticus*, have long been regarded as avirulent organisms. The role of toxins in the development of infections caused by CoNS is still controversial. The objective of this study was to characterize the presence of enterotoxin and cytotoxin genes in *S. epidermidis* and *S. haemolyticus* isolates obtained from blood cultures. Cytotoxin genes were detected by PCR using novel species-specific primers. Among the 85 *S. epidermidis* and 84 *S. haemolyticus* isolates, 95.3% and 79.8%, respectively, carried at least one enterotoxin gene. The most frequent enterotoxin genes were *sea* (53.3%), *seg* (64.5%) and *sei* (67.5%). The *seg* gene was positively associated with *S. epidermidis* (*p* = 0.02), and this species was more toxigenic than *S. haemolyticus*. The *hla*/*yidD* gene was detected in 92.9% of *S. epidermidis* and the *hla* gene in 91.7% of *S. haemolyticus* isolates; *hlb* was detected in 92.9% of the *S. epidermidis* isolates and *hld* in 95.3%. Nosocomial *Staphylococcus epidermidis* and *S. haemolyticus* isolates exhibited a high toxigenic potential, mainly containing the non-classical enterotoxin genes *seg* and *sei*. The previously unreported detection of *hla/yidD* and *hlb* in *S. epidermidis* and *S. haemolyticus* using species-specific primers showed that these hemolysin genes differ between CoNS species and that they are highly frequent in blood culture isolates.

## 1. Background

Coagulase-negative staphylococci (CoNS), including the clinically-significant species *Staphylococcus epidermidis* and *Staphylococcus haemolyticus*, have been well established as significant nosocomial agents of invasive medical device-associated infections [1]. Enterotoxins are well-characterized virulence factors in *Staphylococcus aureus*, and their genes and synthesis have been described in CoNS [2,3], including CoNS causing infections [4,5]. Enterotoxins are superantigens that stimulate the immune system to produce an exaggerated response, causing cytokine release, clonal expansion and clonal deletion of part of these lymphocytes via apoptosis [6]. The release of proinflammatory cytokines is responsible for the rapid onset of high fever, capillary leakage and multiorgan dysfunction. The suddenness and magnitude of cytokine release determine the severity and outcome of the patient [7].

Cytotoxins or hemolysins are important molecules involved in the pathogenesis of *S. aureus*, but their role in CoNS infections is still unknown. α-hemolysin exerts a hemolytic, dermonecrotic and neurotoxic effect [8], while β-toxin possesses phosphorylase activity and high affinity for the cell membrane of different types of cells, causing membrane instability [9]. δ-hemolysin causes lysis of a variety of mammalian cells, including erythrocytes and intracellular structures, such as organelles with an envelope [8]. The δ-toxin gene, *hld*, is located within the RNAIII locus, a transcript of the P3 operon, which acts as an effector of the *agr* quorum sensing system [10]. However, its specific role in the development of staphylococcal infections has not been clearly established. In *S. aureus*, δ-hemolysin is a polypeptide formed by 26 amino acids, while in *S. epidermidis*, it consists of 25 amino acids with high homology to the δ-toxin of *S. aureus* [11].

Few reports have described the presence of cytotoxin-encoding genes and their expression in CoNS [12]. Although there are reports of the presence of the *hla* and *hld* genes encoding α- and δ-hemolysin, respectively, in *S. epidermidis* [13], studies involving other CoNS species that exhibit weak or moderate hemolytic activity in human and bovine erythrocytes and sheep or rabbit blood, particularly *S. haemolyticus*, are sparse [14]. To our knowledge, there is no technique that can efficiently detect the genetic determinants of α- and β-toxins in *S. epidermidis* and *S. haemolyticus*.

The α-hemolysin gene was described in only one strain of *S. epidermidis* (*S. epidermidis* W23144 (GenBank:ACJC01000124.1) and then denoted as “*yidD*”. This gene encodes a protein with 82 amino acids, a membrane protein that possesses α-hemolysin activity [15,16]. Sixty-eight of these amino acids are identical to the hemolytic domain of a protein found in a *S. epidermidis* strain (NCBI: AIR83523.1), called “putative membrane protein insertion efficiency factor” [17].

Therefore, the objective of the present study was to characterize the presence of enterotoxin genes and of the cytotoxin-encoding genes *hla*, *hlb* and *hld* using species-specific primers in *S. epidermidis* and *S. haemolyticus* blood culture isolates.

## 2. Results

### 2.1. Detection of Enterotoxin Genes

A total of 169 isolates were studied, including 85 *S. epidermidis* and 84 *S. haemolyticus*.

**Figure 1:**
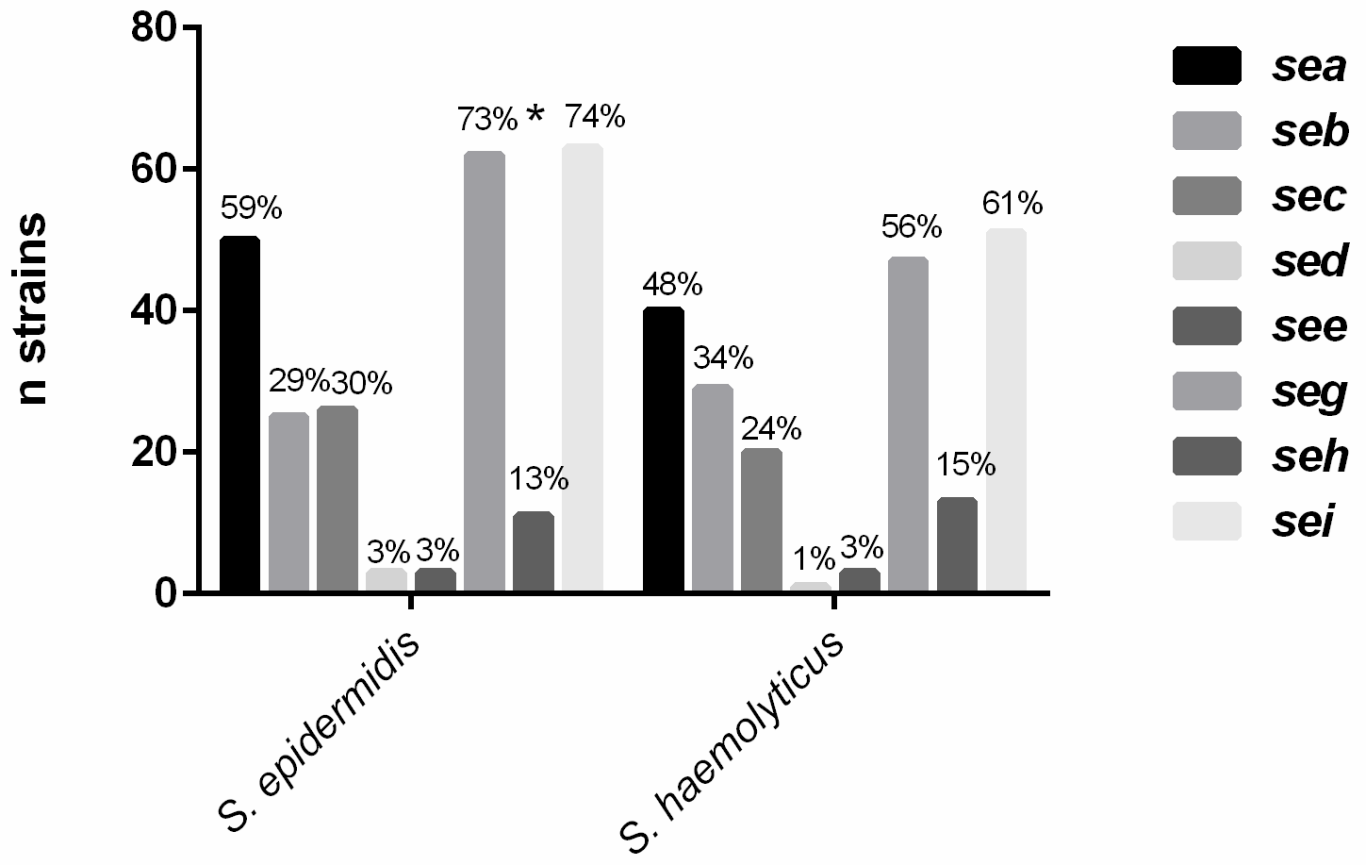
Detection of the enterotoxin genes *sea*–*sei* in *S. epidermidis* and *S. haemolyticus* isolates. * Significantly positive association with *S. epidermidis*.

Figure 1 illustrates the detection of enterotoxin genes in the *S. haemolyticus* and *S. epidermidis* isolates. The proportion of positive isolates was higher for the latter species, except for *seb* and *seh* (34% and 15%, respectively), which were more frequent in *S. haemolyticus*. The *sed* and *see* genes were rarely found (2% and 3%, respectively), while *sei*, *seg* and *sea* were the most frequent genes in both species. Detection of the *seg* gene was significantly associated with *S. epidermidis* (*p* = 0.02).

The *sea* and *seb* genes were concomitantly detected in 20% (*n* = 34) of the isolates, including 17.6% of *S. epidermidis* and 21.4% of *S. haemolyticus*, while *seg* and *sei* were concomitantly present in 52.7% (*n* = 89) (61.2% of *S. epidermidis* and 44% of *S. haemolyticus*). Among the strains studied, 87.6% (*n* = 148) carried at least one enterotoxin gene, including 95.3% (*n* = 81) of the *S. epidermidis* isolates and 79.8% (*n* = 67) of *S. haemolyticus*.

### 2.2. Molecular and Phenotypic Detection of Cytotoxins

The hemolysin genes *hla*/*yidD*, *hlb* and *hld* were detected in *S. epidermidis* using species-specific primers. The *hlb* and *hld* primers of *S. epidermidis* could not identify the β- and δ-toxin genes in *S. haemolyticus*. Novel primers were designed to identify the *hla* gene in *S. haemolyticus*. However, primers for the *S. haemolyticus hlb* and *hld* genes could not be designed, since these genes have not yet been described in that species.

The rate of detection of *hla/yidD* was similar in *S. epidermidis* and *S. haemolyticus* (92.9% and 91.7%, respectively). In *S. epidermidis*, *hlb* was detected in 92.9% of the isolates and *hld* in 95.3%. The *hla* and *hlb* genes were concomitantly present in 89.4% of the *S. epidermidis* isolates. β-toxin was found in 81% of the *S. haemolyticus* isolates and δ-toxin in 40.5%. Thirty percent of the *S. haemolyticus* isolates produced both β- and δ-toxin (Table 1).

**Table 1:** Detection of α-, β- and δ-cytotoxin genes and production.

Organisms (*n*)	hla *		*hlb*		*hld*		α-toxin		β-toxin		δ-toxin	
	*n*	%	*n*	%	*n*	%	*n*	%	*n*	%	*n*	%
*S. epidermidis* (85)	79	92.9	79	92.9	81	95.3	24	28.2	25	29.4	-	-
*S. haemolyticus* (84)	77	91.7	-	-	-	-	70	83.3	68	81	34	40.5
Total (169)	156	92.3	79	92.9	81	95.3	94	55.6	93	55	34	40.5

* *hla*/*yidD* for *S. epidermidis.*

Table 2 shows the comparison between genotypic detection of *hla/yidD* and *hlb* and α- and β-toxin production. The results showed that, while 78% of the *S. haemolyticus* isolates carried *hla* and produced α-toxin, only 28% of the *S. epidermidis* isolates carried the *hla*/*yidD* and *hlb* genes and produced the respective cytotoxins. Discrepancies were observed in the case of five *S. haemolyticus* isolates, which were negative for *hla*, but showed phenotypic production, and one *S. epidermidis* isolate, which was negative for *hlb* and a producer of β-toxin.

**Table 2:** Comparison of the frequency of the *hla* and *hlb* genes and phenotypic production of α- and β-toxins.

*Staphylococcus haemolyticus*				*Staphylococcus epidermidis*						
Genes				Genes			Genes			
Toxin	**hla**+	*hla*−	Total	*hla*+ *	*hla*− *	Total	Toxin	*hlb*+	*hlb*−	Total
	*n*(%)	*n* (%)	*n* (%)	*n* (%)	*n* (%)	*n* (%)		*n* (%)	*n* (%)	*n* (%)
α-Toxin +	65 (78)	5 (6)	70 (84)	24 (28)	0	24 (28)	β-Toxin +	24(28)	1 (1)	25 (29)
α-Toxin −	12 (14)	2 (2)	14 (16)	55 (65)	6 (7)	61 (72)	β-Toxin −	55 (65)	5 (6)	60 (71)
**Total**	77 (92)	7 (8)	84 (100)	79 (93)	6 (7)	85 (100)	**Total**	79 (93)	6 (7)	85 (100)

+, positive; **−**, negative. * *hla*/*yidD*.

The toxin gene profile and phenotypic toxin production of each isolate are shown in Supplemental Table S1.

## 3. Discussion

*Staphylococcus epidermidis* and *S. haemolyticus* are the main CoNS species colonizing the human nose [18], the most common species isolated from blood cultures [19] and are often related to catheter-associated bloodstream infections [20]. In addition to being the main cause of food poisoning, staphylococcal enterotoxins play an important role in pathological processes, such as sepsis, osteomyelitis and respiratory distress syndrome [21]. However, the enterotoxigenic potential of CoNS is controversial.

The present study showed a high frequency of enterotoxin genes in blood culture isolates, with 95.3% of *S. epidermidis* isolates and 79.8% of *S. haemolyticus* isolates carrying at least one toxin gene. The most common classical enterotoxin genes detected were *sea*, *seb* and *sec*, and *seg* and *sei* were the most prevalent among all enterotoxin genes. Another study [22] also showed a higher percentage of *sea*, *seb* and *sec* in CoNS isolated from bovine milk. Furthermore, enterotoxin a production by human CoNS isolates has also been reported [23]. The production of the classical enterotoxins SEA, SEB and SEC by clinical isolates of *S. epidermidis* and of SEC by *S. haemolyticus* has been described [5,24], with a high percentage of isolates producing a combination of two or more toxins [25]. Similar to the present study, the presence of the SEE, SEG, SEH and SEI genes and the production of these enterotoxins have also been reported [26], but studies showing the absence or a low frequency of these genes in CoNS predominate in the literature. These differences between studies may be related to bias in the method used and in the isolates studied, including the number, nature and geographic origin of the strains. Nosocomial isolates may be better equipped with virulence factors obtained by facilitated transfer through selective pressure.

In the present study, 20% of the strains were positive for both *sea* and *seb*. The frequent presence of these two genes in the same bacterium is explained by the fact that they occupy the same chromosome locus [27]. Furthermore, 61.2% of *S. epidermidis* and 44% of *S. haemolyticus* were found to be positive for both *seg* and *sei*. Several studies [28,29] have indicated a systematic association between *seg* and *sei* and a high frequency of these genes in *S. aureus*, which may also occur in CoNS. The concomitant presence of the *seg* and *sei* genes is expected, since these two genes are found in the *egc* cluster, which also contains genes encoding other staphylococcal enterotoxins [30].

*Staphylococcus epidermidis* has been indicated as the CoNS with the highest toxigenic potential in some studies [25]. In fact, this species showed a higher rate of enterotoxin genes compared to *S. haemolyticus* (95.3% *vs*. 79.8%, respectively). A pathogenicity island expressing several enterotoxin genes has been recently described in a clinical *S. epidermidis* isolate [31].

Data regarding the presence of hemolysins and hemolysin genes in CoNS are still sparse. Although 81% of *S. haemolyticus* isolates show β-hemolytic activity and 40% produce δ-toxin, genome sequencing was unable to identify the genes responsible for hemolysis in these species; only the α-hemolysin gene has been demonstrated. Hemolysin primers designed for *S. aureus* [32] and *S. epidermidis*, as well as *hld* primers designed from the *hld* sequence of *S. simulans* (GenBankAccession Number AJ223775.1; forward: AAGGGGGCAATACACATGRC; reverse: CCGAACGCTTCATTTCCGAT), could not detect these genes in *S. haemolyticus*. Huseby *et al.* [33] demonstrated species-specific differences in the β-toxin of *S*. *schleiferi* and *S. epidermidis*, whose proteins showed 72% and 52% homology with *S. aureus* β-toxin, respectively. These differences between β-hemolysins of different CoNS species may be the result of bacterial adaptation to a wide variety of potential hosts [34]. Since the *hlb* and *hld* primers for *S. epidermidis* could not identify these genes in *S. haemolyticus* and the *hlb* and *hld* genes have not yet been described in the latter species, although they are produced as demonstrated by a phenotypic detection method, considerable differences in their sequences may exist, suggesting that these toxins have a distinct structure and, consequently, different functions in CoNS species.

To our knowledge, this is the first study to detect the *hla* gene using specific primers for *S. epidermidis* and *S. haemolyticus*. The gene used for the primer design of strain *S. epidermidis* W23144 had been denoted in GenBank as “α-hemolysin” until June 2013. On that date, the authors altered the denotation of this gene to “*yidD*” and classified it as a membrane protein. According to previous studies, some members of the yidD family were annotated as hemolysins, which resulted from the unpublished observation reported in GenBank L36462 that the *hlyA* gene, which is homologous to *yidD* of *Aeromonas hydrophila*, possesses α-hemolysin activity [15,16]. Some databases show that *yidD* is orthologous to the proteins with hemolytic function SE1462 of *S. epidermidis* ATCC 12228 and SERP1356 of *S. epidermidis* RP62A (http://www.xbase.ac.uk/genome/buchnera-aphidicola-str-sg-schizaphis-graminum/NC_004061/BUsg015;yidD/super/orthologues).

The α-toxin/*yidD*-encoding gene was found in 92.9% of the *S. epidermidis* isolates and the *hla* gene in 91.7% of the *S. haemolyticus* isolates, while *hlb* was detected at the same frequency (92.9%) in *S. epidermidis*. On the other hand, another study [13] detected *hla* in only 20% of *S. epidermidis* isolates and the absence of *hlb* in all strains. β-toxin has been described in 75% of CoNS and α-hemolysis in 57% [35]. Nataro *et al.* [36] observed 61% of positivity for β-toxin in CoNS, while in the present study, β-hemolysin production was observed in 81% of the *S. haemolyticus* isolates. Moraveji *et al.* [37] observed double the frequency of hemolysin genes and production in human strains compared to animal strains. The importance of *hlb* and β-toxin is due to the ability of this protein to promote the escape of bacteria from the host immune system and to its involvement in nutrient uptake [33], permitting survival of the pathogen.

The divergence in the *hld* gene is so high among species that it cannot be amplified in some CoNS [12]. This diversity is demonstrated by the fact that the partial identity of this toxin gene between *S. aureus* and *S. epidermidis* is only 83% [12]. The same may apply to *S. haemolyticus* and may explain the lack of amplification of this gene by *S. epidermidis* primers in the present study. δ-hemolysin is encoded by regulatory RNAIII in *S. aureus* associated with the *agr* system [38], a system described in several staphylococcal species, including *S. epidermidis* and *S. haemolyticus* [39,40]. In the present study, the *hld* gene was detected in 95.3% of the *S. epidermidis* isolates, and δ-hemolysin was produced by 40.5% of the *S. haemolyticus* isolates. According to Gemmel [41], δ-hemolysin is more frequently expressed by CoNS isolated from clinically-important infections compared to inapparent human infections.

Despite the high frequency of the *hla* gene observed in the present study in *S. epidermidis* and *S. haemolyticus*, phenotypic production of the toxin encoded by *hla* seems to be more frequent in the latter species, with most *hla*-positive *S. haemolyticus* isolates (85%) expressing α-toxin. In contrast, despite the high frequency of *hla*/*yidD* and *hlb* in *S. epidermidis*, less than one-third (30%) of the isolates carrying these genes also expressed them. The absence of the gene and the presence of the toxin observed in five *S. haemolyticus* isolates and in one *S. epidermidis* isolate might be related to mutations in the sequences of these genes, such as insertion sequences that interfere with the amplification of the gene by PCR.

One limitation of the present study is the fact that the prevalence of the toxigenic genes is not equivalent to the prevalence of the expression of these genes. However, expression was demonstrated in this study by hemolysis on blood agar. Further studies using other methods to evaluate the expression of these genes, such as Western blotting, are needed. Furthermore, genome sequencing of some of these positive strains will be important to identify these genes in the genomes of *S. epidermidis* and *S. haemolyticus*.

## 4. Material and Methods

### 4.1. Isolates

The strains were isolated from blood cultures of inpatients admitted to the University Hospital of the Botucatu Medical School (Hospital das Clínicas, Faculdade de Medicina de Botucatu (HC-FMB)), Paulista State University (Universidade Estadual Paulista (UNESP)), Botucatu Campus, between 2000 and 2011. Only one isolate per patient was included in the study. The strains were isolated as described by Koneman *et al.* [42].

### 4.2. Species Identification

The genus *Staphylococcus* was identified as described by Koneman *et al.* [42]. *Staphylococcus epidermidis* and *S. haemolyticus* were identified by the simplified method proposed by Cunha *et al.* [43]. Species identification was genetically confirmed by PCR amplification of the 16S-23S internal transcribed spacer (ITS) region as described by Couto *et al.* [44] after DNA extraction with the Illustra kit (GE Healthcare, Little Chalfont, Buckinghamshire, UK). The following international reference strains were used as controls: *S. epidermidis* (ATCC 12228), *S. epidermidis* (ATCC 35983) and *S. haemolyticus* (ATCC 29970).

### 4.3. Detection of Enterotoxin Genes

PCR for the detection of enterotoxin genes was performed using the primers and parameters described by Johnson *et al.* [45] and Cunha *et al.* [4]. International reference strains were included in all reactions as positive (*S. aureus* American Type Culture Collection—ATCC 13565 (*sea*), ATCC 14458 (*seb*), ATCC 19095 (*sec*), ATCC 23235 (*sed*), ATCC 27664 (*see*), ATCC 51811 (*seh*), *S. aureus* Food Research Institute—FRI 361 (*seg* and *sei*)) and negative (*S. xylosus* ATCC 29971) controls. The primer sequences are shown in Table 3.

**Table 3:** Sequence of primers and amplicon size.

Name	Product	Sequence	Reference	Amplicon Size (bp)
*sea-1*	Enterotoxin A	TTGGAAACGGTTAAAACGAA	[29]	120
*sea-2*		GAACCTTCCCATCAAAAACA		
*seb-1*	Enterotoxin B	TCGCATCAAACTGACAAACG	[29]	478
*seb-2*		GCAGGTACTCTATAAGTGCC		
*sec-1*	Enterotoxin C	GACATAAAAGCTAGGAATTT	[29]	257
*sec-2*		AAATCGGATTAACATTATCC		
*sed-1*	Enterotoxin D	CTAGTTTGGTAATATCTCCT	[29]	317
*sed-2*		TAATGCTATATCTTATAGGG		
*see-1*	Enterotoxin E	CAAAGAAATGCTTTAAGCAATCTTAGGCCAC	[29]	170
*see-2*		CTTACCGCCAAAGCTG		
*seg-1*	Enterotoxin G	AATTATGTGAATGCTCAACCCGATC	[36]	642
*seg-2*		AAACTTATATGGAACAAAAGGTACTAGTTC		
*sei-1*	Enterotoxin H	CAATCACATCATATGCGAAAGCAG	[36]	376
*seh-2*		CATCTACCCAAACATTAGCACC		
*sei-1*	Enterotoxin I	CTCAAGGTGATATTGGTGTAGG	[36]	576
*sei-2*		AAAAAACTTACAGGCAGTCCATCTC		
*hla/yidD_epid-1*	α-hemolysin/yidD	TTTCKCCACTTACACCMCC	This study	160
*hla/yidD_epid-2*		GGAACAGGATCAAAGCCACCT		
*hlb_epid-1*	β-hemolysin	TGGTGGCGTTGGTATTGTGA	This study	541
*hlb_epid-2*		ACCCCAAGATTTCACGGACC		
*hla_haem-1*	α-hemolysin	TGGGCCATAAACTTCAATCGC	This study	72
*hla-haem-2*		ACGCCACCTACATGCAGATTT		
*hld-epid-1*	δ-hemolysin	ATGGCAGCAGATATCATTTC	[30]	444
*hld-epid-2*		CGTGAGCTTGGGAGAGAC		

### 4.4. Detection of Hemolysin Genes

The δ-hemolysin gene, *hld*, was detected using the primers and parameters described by Marconi *et al.* [46].

The *hla*/*yidD* gene was detected in *S. epidermidis* isolates using primers designed with NCBI-PrimerBlast, 2008 and sequences of the strain *S. epidermidis* W23144 (GenBank: ACJC01000124.1) (hla/yidD_epid). The *hla* gene was detected in *S. haemolyticus* isolates using primers designed with PrimerBlast and the sequence of the strain JCSC1435 (NCBI: NC_007168.1) (hla_haem). Primers for the *hlb* gene in *S. epidermidis* were designed using PrimerBlast and the sequence of *S. epidermidis* RP62A (NCBI:NC_002976.3) (hlb_epid). Primers for the *hlb* gene in *S. haemolyticus* could not be designed, since this gene has not been described in that species in the NCBI-GenBank database. The reaction mixture contained 2.0 U Taq polymerase, 1× PCR buffer containing 0.75 mM MgCl_2_, 100 µM triphosphate deoxyribonucleotides, 1 M of each primer and 150 ng nucleic acid. The PCR conditions were as follows: for *hla*, one step at 94 °C for 4 min; 35 cycles at 94 °C for 1 min, 60 °C for 1 min, 72 °C for 1 min and 72 °C for 5 min; for *hlb*, one step at 94 °C for 4 min, 35 cycles at 94 °C for 1 min, 60 °C for 1 min, 72 °C for 1.5 min and 72 °C for 6 min. Reference strains were included in all reactions: *hla/yidD*_epid: *S. epidermidis* ATCC 12228; *hla*_haem: *S. haemolyticus* ATCC 29970; *hlb*_epid: *S. epidermidis* ATCC 12228. The primer sequences are shown in Table 3.

### 4.5. Phenotypic Production of β-and δ-Cytotoxins

The production of α-toxin was determined on blood agar plates containing 5% rabbit blood incubated at 37 °C for 24 h. A positive result was indicated by the formation of hemolysis zones around the isolated colonies.

β- and δ-toxin production in *S. haemolyticus* isolates was detected as described by Hébert and Hancock [47]. β-hemolysis was observed by the presence of a zone with incomplete hemolysis on a sheep blood agar plate incubated at 37 °C for 24 h and then overnight at 4 °C [48]. The presence of δ-toxin was verified by the presence of synergism with β-hemolysin of *S. aureus* ATCC 25923. For this purpose, the isolate was streaked perpendicular to the *S. aureus* strain on a sheep blood agar plate. The plate was incubated at 37 °C for 24 h, and δ-toxin production was observed by the formation of an arrowhead-shaped zone of hemolysis [47].

### 4.6. Statistical Analysis

The chi-square test was used to verify the association between variables, adopting a level of significance <0.05.

## 5. Conclusions

The clinical isolates of *S. epidermidis* and *S. haemolyticus* exhibited a high toxigenic potential, producing especially enterotoxins G and I. The use of novel species-specific primers for *hla/yidD* and *hlb* of *S. epidermidis* and for *hla* of *S. haemolyticus* revealed a high frequency of these genes in nosocomial isolates of these species. The findings demonstrate an important role of these cytotoxin genes in the establishment of these species and possibly in the development of infections caused by CoNS.

## Supplementary Materials

Supplementary materials can be accessed at: http://www.mdpi.com/2072-6651/7/9/3688/s1.
