# Supplementary material for: Staphylococcus epidermidis and Staphylococcus haemolyticus: Molecular Detection of Cytotoxin and Enterotoxin Genes
Source: Toxins (Basel). 2015 Sep 14;7(9):3688–99. doi: 10.3390/toxins7093688 (PMC4591658; doi:10.3390/toxins7093688)
Supplement: Supplementary file 1 [file toxins-07-03688-s001.pdf]

Supplementary Information

Table S1. Profile of isolates positive for the *sea–sei*, *hla* *, *hlb* and *hld* genes and phenotypic production of α-, β- and δ-toxins.

Isolate	*sea*	*seb*	*sec*	*sed*	*see*	*seg*	*seh*	*sei*	*hla* *	*hlb*	*hld*	α-toxin	β-toxin	δ-toxin
Staphylococcus haemolyticus														
H-310/01	+	−	−	−	+	+	−	+	+	N	N	+	+	−
H-2620/01	+	+	+	−	−	+	−	+	+	N	N	−	+	−
H-30006/03	+	+	+	−	−	+	+	+	+	N	N	+	+	−
H-31135/04	−	−	−	−	−	−	+	+	+	N	N	+	+	−
H-31158/04	−	−	−	−	−	+	+	+	+	N	N	+	+	−
H-2504/05	+	+	+	−	−	+	−	+	+	N	N	+	−	−
H-2527/05	+	−	+	−	−	+	−	+	+	N	N	+	+	+
H-1009/06	−	+	−	−	−	+	−	+	+	N	N	+	+	+
H-1347/06	+	+	+	−	−	+	+	+	+	N	N	−	+	+
H-2098/06	+	−	−	−	−	+	−	+	+	N	N	+	+	+
H-1764/08	+	+	−	+	−	+	−	+	+	N	N	+	+	+
H-39/09	+	−	−	−	−	+	−	+	+	N	N	+	+	+
H-180/09	+	+	+	−	−	+	+	+	+	N	N	+	+	−
H-384/09	+	−	+	−	−	+	−	−	+	N	N	−	+	−
H-560/09	+	−	−	−	−	+	−	+	+	N	N	+	+	−
H-892/09	+	−	+	−	−	+	+	+	+	N	N	+	+	−
H-1271/09	+	+	−	−	−	+	+	−	+	N	N	+	+	+
H-3157/09	+	+	−	−	+	+	−	−	+	N	N	+	+	+
H-3438/09	−	−	−	−	−	−	−	−	+	N	N	+	−	+
H-5732/09	+	+	−	−	−	+	−	−	+	N	N	+	+	−
H-6067/09	−	−	−	−	−	+	−	+	+	N	N	+	+	+
H-2989/09	−	−	−	−	−	+	−	+	+	N	N	+	+	−
H-4741/08	+	−	+	−	−	−	−	+	+	N	N	+	−	+
H-4913/08	+	+	+	−	−	+	−	−	+	N	N	−	+	+

Table S1. *Cont.*

Isolate	*sea*	*seb*	*sec*	*sed*	*see*	*seg*	*seh*	*sei*	*hla* *	*hlb*	*hld*	α-toxin	β-toxin	δ-toxin
S. haemolyticus														
H-6025(1)/09	−	−	+	−	−	−	−	−	+	N	N	+	+	+
H-6921/10	−	−	−	−	−	−	−	−	+	N	N	+	+	+
H-6435/10	−	−	−	−	−	−	−	+	+	N	N	+	+	+
H-7220/11	+	−	+	−	+	+	−	−	+	N	N	+	+	−
H-7245/11	−	−	−	−	−	−	−	+	+	N	N	+	+	+
H-5083/10	−	−	−	−	−	+	−	−	+	N	N	+	+	+
H-113/08	−	−		−	−	−	−	+	−	N	N	+	+	−
H-4798/05	−	−	−	−	−	−	−	−	+	N	N	+	+	+
H-2322/11	−	−	−	−	−	−	−	−	+	N	N	+	+	+
H-4949/05	−	−	−	−	−	−	−	−	+	N	N	+	+	−
H-4936/05	−	−	−	−	−	−	−	−	+	N	N	+	+	−
H-2813-4/10	+	−	−	−	−	+	−	−	+	N	N	+	+	−
H-2923/11	+	−	−	−	−	−	−	−	+	N	N	+	−	−
H-30006/10	+	−	−	−	−	−	−	−	+	N	N	+	−	+
H-2741/10	−	−	−	−	−	−	−	−	+	N	N	−	+	+
H-8171/11	−	−	−	−	−	−	−	−	+	N	N	+	+	−
H-6836-7/10	−	−	−	−	−	−	+	−	+	N	N	−	+	−
H-4550/10	−	−	−	−	−	−	−	−	+	N	N	−	+	+
H-5470/10	−	−	−	−	−	−	−	−	+	N	N	+	−	+
H-7323/11	+	−	−	−	−	−	−	−	+	N	N	+	−	+
H-7902/11	−	−	−	−	−	−	−	−	+	N	N	+	+	+
H-6421/10	−	−	−	−	−	+	−	−	+	N	N	−	+	+
H-4684/10	−	−	−	−	−	−	−	−	+	N	N	−	+	−
H-987/08	−	+	+	−	−	−	−	+	+	N	N	+	+	−
H-874/08	−	+	−	−	−	−	−	+	+	N	N	+	+	−
H-5433/08	+	+	−	−	−	−	+	+	+	N	N	+	+	−

Table S1. *Cont.*

Isolate	*sea*	*seb*	*sec*	*sed*	*see*	*seg*	*seh*	*sei*	*hla* *	*hlb*	*hld*	α-toxin	β-toxin	δ-toxin
							S. haemolyticus							
H-6153/09	−	+	−	−	−	+	−	+	+	N	N	+	+	−
H-2420/08	−	−	−	−	−	+	−	+	+	N	N	+	+	−
H-2437/08	+	+	−	−	−	+	−	+	+	N	N	+	+	−
H-2346-47/08	+	+	+	−	−	−	−	+	+	N	N	+	+	−
H-1674/08	−	−	−	−	−	−	−	−	+	N	N	+	+	−
H-1590/09	−	+	−	−	−	+	−	+	+	N	N	+	+	−
H-4078/08	−	−	−	−	−	+	−	+	+	N	N	−	−	−
H-5341/08	+	−	−	−	−	+	−	+	+	N	N	+	−	−
H-1557/08	−	−	−	−	−	+	−	+	+	N	N	+	+	−
H-1667/07	+	+	−	−	−	+	−	+	+	N	N	+	+	+
H-3160/07	−	+	−	−	−	+	−	+	+	N	N	+	+	−
H-4564/05	−	+	−	−	−	+	−	+	+	N	N	+	+	+
H-764/07	−	+	+	−	−	+	−	+	−	N	N	+	+	−
H-2218/06	+	−	−	−	−	+	−	+	−	N	N	−	−	−
H-30852/05	+	+	−	−	−	+	−	+	−	N	N	+	+	+
H-3631/05	+	+	−	−	−	+	−	−	+	N	N	+	+	−
H-5557/07	−	+	+	−	−	+	−	+	+	N	N	−	−	−
H-5001/07	−	+	−	−	−	+	+	+	+	N	N	+	+	−
H-3518/07	−	−	+	−	−	+	−	+	+	N	N	+	−	+
H-3511/07	−	+	+	−	−	+	−	+	+	N	N	+	−	+
H-5759/07	+	−	−	−	−	+	+	+	+	N	N	+	+	−
H-5973/07	+	+	−	−	−	+	−	+	+	N	N	+	+	−
H-3657/07	+	+	−	−	−	+	−	+	+	N	N	+	+	−
H-3131/02	+	−	−	−	−	−	−	+	+	N	N	+	+	+
H-1148/02	+	−	−	−	−	+	−	+	−	N	N	−	−	−
H-3374/11	+	−	−	−	−	−	−	+	−	N	N	+	−	+

Table S1. *Cont.*

Isolate	*sea*	*seb*	*sec*	*sed*	*see*	*seg*	*seh*	*sei*	*hla* *	*hlb*	*hld*	α-toxin	β-toxin	δ-toxin
S. haemolyticus														
H-6082/11	−	−	−	−	−	−	−	−	−	N	N	+	−	−
H-3257/11	−	−	+	−	−	−	+	+	+	N	N	+	+	−
H-4057/11	+	−	−	−	−	−	−	+	+	N	N	+	+	−
H-4112/11	+	−	+	−	−	−	+	+	+	N	N	+	+	−
H-4221/11	+	−	−	−	−	−	−	−	+	N	N	−	+	+
H-5231/11	−	−	−	−	−	−	−	−	+	N	N	+	+	−
H-5465/11	−	−	−	−	−	−	−	−	+	N	N	+	+	−
H-5996/11	−	−	−	−	−	−	−	−	+	N	N	+	+	−
Staphylococcus epidermidis														
H-426/00	+	−	+	−	+	−	−	+	−	−	+	−	−	N
H-480/00	+	−	+	−	−	−	−	+	−	−	+	−	−	N
H-1906/00	−	−	−	−	−	−	−	−	+	+	+	+	+	N
H-368/01	−	+	+	−	−	+	−	+	+	−	+	−	−	N
H-852/01	+	−	−	−	−	−	−	+	+	+	−	−	+	N
H-1022/01	−	−	−	−	−	−	−	+	+	+	+	+	−	N
H-1243/01	+	−	−	−	−	−	−	+	+	+	+	−	−	N
H-1244/01	−	−	−	−	−	+	−	−	+	+	+	−	−	N
H-1411/01	−	+	−	−	−	+	−	+	+	+	+	−	−	N
H-1469/01	+	−	−	−	−	+	−	+	+	+	+	+	−	N
H-1472/01	−	+	−	−	−	+	+	+	+	+	+	−	−	N
H-1532/01	+	−	+	−	−	+	−	+	+	−	+	−	−	N
H-1547/01	−	+	+	−	−	+	−	+	+	+	+	−	+	N
H-1553/01	−	+	−	−	−	+	−	+	+	+	+	+	−	N
H-1575/01	+	+	+	−	−	+	−	+	+	+	+	−	−	N
H-271/02	−	−	+	−	−	+	−	+	+	+	+	−	−	N
H-273/02	+	−	−	−	−	+	−	+	+	+	+	−	−	N

Table S1. *Cont.*

Isolate	*sea*	*seb*	*sec*	*sed*	*see*	*seg*	*seh*	*sei*	*hla* *	*hlb*	*hld*	α-toxin	β-toxin	δ-toxin
S. epidermidis														
H-283/02	+	−	−	−	−	+	−	+	+	+	+	+	+	N
H-817/02	+	−	+	−	−	+	−	+	+	+	+	−	+	N
H-161/03	−	+	−	−	−	+	−	−	+	+	+	+	−	N
H-192/03	−	+	+	−	−	+	−	−	+	+	+	−	−	N
H-239/03	−	−	−	−	−	+	−	+	+	+	+	+	−	N
H-298/03	−	+	+	−	−	+	−	+	+	+	+	−	−	N
H-30001/04	+	+	+	−	−	+	−	+	+	+	+	−	−	N
H-30007/04	+	+	−	−	−	+	−	+	+	+	+	−	−	N
H-30053/04	−	−	+	−	−	−	−	+	+	+	+	−	−	N
H30146/04	+	+	−	−	−	+	−	+	+	+	+	−	+	N
H-30150/04	+	+	−	−	−	+	−	+	+	+	+	−	+	N
H-1402/05	−	−	−	−	−	+	−	+	+	+	+	−	+	N
H-1765/05	+	−	−	−	−	+	+	+	+	+	+	+	+	N
H-1915/05	+	−	−	−	−	+	−	+	−	+	−	−	−	N
H-2120/05	+	−	−	−	−	+	−	+	+	+	+	−	−	N
H-2202/05	−	−	−	−	−	+	−	−	+	+	+	+	+	N
H-2533/05	−	−	−	−	−	+	+	+	−	+	−	−	−	N
H-2696/05	−	−	+	−	−	+	−	+	+	+	+	−	−	N
H-2975/05	−	−	−	−	−	+	−	+	+	+	+	−	+	N
H-30823/05	+	+	−	−	−	+	−	+	+	+	+	−	−	N
H-30888/05	−	−	−	−	−	−	−	+	+	+	+	+	+	N
H-31/06	+	−	−	−	−	−	−	+	+	+	−	−	−	N
H-280/06	+	−	−	−	−	+	−	+	+	+	+	−	−	N
H-923/06	+	−	+	−	−	+	+	+	+	+	+	+	+	N
H-1054-55/06	+	−	−	−	−	+	+	+	+	+	+	−	−	N
H-1263/06	+	−	+	−	−	−	−	−	+	+	+	−	−	N

Table S1. *Cont.*

Isolate	*sea*	*seb*	*sec*	*sed*	*see*	*seg*	*seh*	*sei*	*hla* *	*hlb*	*hld*	α-toxin	β-toxin	δ-toxin
S. epidermidis														
H-1285/06	+	−	+	−	−	−	+	+	+	+	+	−	−	N
H-1793/06	−	−	−	−	−	+	−	+	+	+	+	−	−	N
H-2210/06	−	−	−	−	−	−	−	+	+	+	+	+	+	N
H-2327/06	+	−	−	−	−	+	−	+	+	+	+	+	−	N
H-2619/06	−	−	−	−	−	−	−	+	+	+	+	+	−	N
H-9/07	−	−	−	−	−	+	−	+	+	+	+	+	+	N
H-133/07	+	−	−	−	−	+	−	−	+	+	+	+	+	N
H-248/07	+	−	−	−	−	+	+	+	+	+	+	+	+	N
H-351/07	+	−	−	−	−	+	−	+	+	+	+	−	−	N
H-655/07	−	−	−	−	+	+	−	+	+	+	+	−	+	N
H-5519/07	+	−	+	−	−	+	−	+	+	+	+	−	−	N
H-5566/07	+	+	−	−	−	+	−	+	+	+	+	−	−	N
H-5693/07	+	+	−	−	−	+	−	+	+	+	+	−	−	N
H-10/08	−	+	−	−	−	−	−	−	+	+	+	+	+	N
H-27/08	+	−	−	−	−	+	−	+	+	+	+	−	−	N
H-187/08	+	+	−	+	−	−	−	−	+	+	+	+	−	N
H-693/08	+	−	+	−	−	+	−	+	+	+	+	+	−	N
H-1189/08	+	−	−	−	−	+	−	+	+	+	+	−	−	N
H-1200/08	−	−	−	−	−	−	−	−	+	+	+	−	−	N
H-1801/08	−	−	+	−	−	−	−	−	+	+	+	−	−	N
H-1805/08	+	−	−	+	−	+	−	+	+	+	+	−	−	N
H-1896/08	−	−	−	−	−	−	−	−	+	+	+	−	−	N
H-2123/08	−	+	−	−	−	−	−	−	+	+	+	−	+	N
H-1640/08	+	+	−	−	−	+	−	+	+	+	+	−	−	N
H-49/09	+	+	−	−	−	+	−	+	−	+	+	−	−	N
H-107/09	+	+	−	−	−	+	−	+	+	+	+	−	−	N

Table S1. *Cont.*

Isolate	*sea*	*seb*	*sec*	*sed*	*see*	*seg*	*seh*	*sei*	*hla* *	*hlb*	*hld*	α-toxin	β-toxin	δ-toxin
S. epidermidis														
H-281/09	+	−	+	−	−	+	−	+	+	+	+	−	−	N
H-611/09	+	+	−	+	−	+	−	+	+	+	+	−	−	N
H-641/09	+	+	−	−	−	+	−	+	+	+	+	−	+	N
H-642/09	+	+	−	−	+	+	−	+	+	+	+	−	−	N
H-700/09	+	−	+	−	−	+	−	+	+	+	+	−	−	N
H-702/09	+	−	−	−	−	+	−	+	+	+	+	−	−	N
H-5019/09	+	−	−	−	−	+	−	+	−	−	+	−	−	N
H-333/10	+	−	−	−	−	−	−	−	+	+	+	−	−	N
H-834/10	−	−	−	−	−	−	−	−	+	+	+	−	+	N
H-3657/10	−	−	−	−	−	+	+	−	+	+	+	−	+	N
H-5344/10	+	−	+	−	−	+	−	−	+	+	+	+	−	N
H-4651/10	+	−	+	−	−	+	−	−	+	+	+	+	−	N
H-3534/10	−	−	+	−	−	+	−	−	+	+	+	−	−	N
H-3721/10	−	−	−	−	−	+	+	−	+	+	+	+	+	N
H-2647/10	+	−	+	−	−	−	+	−	+	−	+	−	+	N
H-3700/10	−	−	+	−	−	−	+	−	+	+	+	+	−	N

+, positive; −, negative; N, not performed. * *hla*/*yidD* for *S. epidermidis*.
